# Magnetic resonance spectroscopic study of radiogenic changes after radiosurgery of cerebral arteriovenous malformations with implications for the differential diagnosis of radionecrosis

**DOI:** 10.1186/1748-717X-8-54

**Published:** 2013-03-07

**Authors:** Jan Boström, Dariusch R Hadizadeh, Wolfgang Block, Winfried Willinek, Hans H Schild, Frank Träber

**Affiliations:** 1 Department of Neurosurgery, University of Bonn Medical Center, Sigmund-Freud-Str. 25, Bonn 53105, Germany; 2 Department of Radiology, University of Bonn Medical Center, Sigmund-Freud-Str. 25, Bonn 53105, Germany; 3 Department of Radiosurgery and Stereotactic Radiotherapy, Mediclin Robert Janker Clinic, Villenstrasse 8, Bonn 53129, Germany

**Keywords:** Radiation necrosis, Radiation injury, Magnetic resonance spectroscopy, Magnetic resonance angiography, Radiosurgery

## Abstract

**Background:**

The incidence of radionecrosis after radiosurgery is 5–20%. That radionecrosis after radiosurgery may be confused with a malignant tumor is a known phenomenon and problem.

**Methods:**

Three similarly treated patients with cAVM, 1 patient with symptomatic radionecrosis and 2 patients with normal post-radiation MRI changes, were selected and studied in detail with magnetic resonance imaging (MRI), magnetic resonance angiography (MRA), and magnetic resonance spectroscopy (MRS). 2 cAVM were located in eloquent locations and were classified as Spetzler-Martin grade (SM) III such that interdisciplinary radiosurgery was recommended; a third patient with a left frontal SM II cAVM refused surgery. 1 patient was male, and 2 were female. The patient’s ages ranged from 38 to 62 years (median, 39 years). The nidus volume (= planning target volume = PTV) ranged from 2.75 to 6.89 ccm (median, 6.41 ccm). The single dose was 20 Gy at the isocenter of the PTV encompassing the 80 – 90% isodose. The median follow-up period was 20 months (range, 16 – 84 months). Toxicities were evaluated with the Common Terminology Criteria (CTC) for adverse events version 3.0.

**Results:**

No patient suffered a bleeding from cAVM during the study period. A complete nidus occlusion was shown in all patients with time-resolved MRA. All patients showed radiogenic MRI changes, 1 patient showed excessive radionecrosis. This patient was oligosymptomatic and under temporary corticoid therapy symptoms resolved completely.

Following patterns associated with radionecrosis in the MRS studies were identified in our collective:

• 2D spectroscopic imaging (2D-SI) revealed much lower concentrations of metabolites in the lesion as compared to contralateral healthy tissue in all patients.

• Whereas regions with regular post-radiosurgery effects showed almost normal levels of Cho and a Cho/Cr ratio < 2.0, regions with radionecrosis were characterized by increased lipid levels and a Cho/Cr ratio > 2.0 in conjunction with decreased absolute levels of all metabolites, especially of Cr and NAA.

**Conclusions:**

MRS is an increasingly valuable tool for the differential diagnosis of radiation reactions. Specific patterns of MRS spectra in radionecrosis were identified; in synopsis with clinical parameters, these changes have to be taken into account to avoid misdiagnosis.

## Background

Radiation injury or radiation necrosis is an often delayed, either calculated reaction or complication after cranial radiotherapy (RT). That radionecrosis after RT may be confused with a malignant tumor on magnetic resonance imaging (MRI) is a known phenomenon and problem [1]. The incidence of radiation injury after standard fractionated external beam radiation therapy of 72 Gy is estimated to be 5% [2]. The incidence after stereotactic radiosurgery (SRS) is reported to be 5–20%, depending on the dose, volume and localization [3,4].

Non-invasive imaging methods, such as magnetic resonance spectroscopy (MRS), have been used to distinguish radiation injury from tumor. MRS provides metabolic and chemical information about brain lesions. It essentially offers in vivo chemical patterns of tissue that reflect ongoing metabolic activity in target lesions; e.g., cellular necrosis, gliotic changes, inflammation or proliferation of neoplastic tissue. Single- and multivoxel techniques may be used when performing MRS, and a variety of metabolic markers can be measured. Choline (Cho) levels reflect the membrane metabolism that is associated with cellular turnover. N-acetyl-aspartate (NAA) functions as a neuronal cell marker, while total creatine (creatine + phosphocreatine, Cr) indicates cellular bioenergetics. Lipid and lactate peaks are present in necrotic tissue and reflect cell membrane destruction and anaerobic metabolism. Results from MRS may be comparable to those from FDG-PET or SPECT [5]. Although there have been some promising retrospective studies [6-8], the true value of MRS in the differentiation of radiation injury from neoplastic tissue remains to be clarified [9]. Furthermore, the majority of those studies derived their data from the radiotherapy of tumors (gliomas or brain metastases), so that the diagnostic utility of MRS after RT of other lesions like arteriovenous malformations (AVMs) - as in the present study - remains less clear.

AVMs are congenital vascular anomalies consisting of a complex web of feeding arteries and draining veins, linked by an abnormal capillary bed, the so-called nidus. Clinically, AVMs are recognized mostly because of intracranial hemorrhage or epilepsy, although they may remain silent over a long time. Because of their life-long cumulative risk of bleeding - approximately 2% to 4% per year - therapeutic strategies are surgical resection or complete obliteration either by radiosurgery or endovascular intervention, and sometimes a combination of these methods is necessary [10].

Radiosurgery can cause occlusion of the AVM nidus in 60 – 90% of cases over a 1 – 2 year period following treatment, provided that the nidus is less than 2 – 3 cm in diameter. MRI changes post-SRS for AVMs occur in up to 30% of cases, depending on the treatment volume and dose administered. Delayed symptomatic radionecrosis occurs in up to 9% of cases, with the majority being transient [11].

In contrast to former MRS studies, we extensively and exclusively investigated non-tumor patients with vascular malformations (AVMs of the brain) particularly avoiding overlaps by tumor necrosis, tumor progression or an infiltrative zone (e.g. in gliomas). With minor restrictions this allows for the unique opportunity to selectively study MRS changes in radionecrosis after radiosurgery.

## Methods

### Patients

Three similarly treated patients with AVMs (stereotactic radiosurgery after endovascular therapy) were collected from the total pool of AVM patients from the department of the first author and were studied in detail with MRI, time-resolved magnetic resonance angiography (MRA) [12], and MRS. This included one patient with symptomatic radionecrosis and two patients with normal post-radiation MRI changes.

The patient with radionecrosis received additional MRI and MRS studies to exclude a secondary malignancy.

2 AVMs were located in an eloquent location and were classified as Spetzler-Martin grade (SM) III with a markedly increased risk in case of surgery, one patient with a left frontal SM II AVM refused surgery. The patients were therefore alternatively treated with radiosurgery, in all cases after multiple (2 – 3) partial embolizations.

Complete nidus occlusion (17 months after radiosurgery) was shown by time-resolved MRA and confirmed using conventional digital subtraction angiograpy (DSA) in one patient. In the other two patients (16 months respectively 7 years after irradiation) nidus occlusion could also be demonstrated by time-resolved MRA as an alternative to conventional DSA, which was refused in both cases. In 2 patients the diagnosis of the AVM was an incidental finding, the other patient reported dizziness and headache, which could not definitely be related to the AVM. No patient has suffered an AVM bleeding until now. The patients and AVM characteristics are summarized in Table 1.

**Table 1:** Summary of patients’ data with special respect to Spetzler-Martin grade (SM), AVM size (maximal diameter in cm), Localization, Eloquence (+/−), Presentation prior to radiosurgery, Venous drainage (D= deep, S= superficial), Rupture before treatment (+/−) and additional treatment modality (E=Embolization)

**No**	**Age**	**Sex**	**Presentation**	**Rupture**	**SM**	**AVM size (cm)**	**Localization**	**Eloquence**	**Venous drainage**	**Pre-SRS treatment**
1	64	F	None	-	3	2.4	Left frontal operculum	+	S + D	3 x E
2	39	M	Headache	-	2	2.7	F2 and Left frontal white matter	+	S	2 x E
3	38	F	None	-	3	3.0	Fronto-lateral left	-	S+D	3 x E

M=male, F=female.

### Radiosurgical treatment

All three patients with AVM were treated with SRS during December 2004 and April 2009 using the the Novalis® radiosurgical system (BrainLAB®, Feldkirchen, Germany) at the Department of Radiosurgery and Stereotactic Radiotherapy, Mediclin Robert Janker Clinic Bonn, Germany. The used Novalis® system consists of a 6 MV LINAC (Varian® Medical Systems, Palo Alto, CA, USA) equipped with a micro-multi-leaf-collimator with a leaf thickness of 3 mm and a robotic couch (Varian® Medical Systems, Palo Alto, CA, USA). After sharp fixation in the stereotactic frame (BrainLAB®, Feldkirchen, Germany) patients underwent first stereotactic angiography and then a planning computed tomogram (CT) using the same localizer. Scanning was performed with 1–3 mm slice thickness without gap for treatment planning using a CT-based 3D treatment planning system. After stereotactic registration and image fusion with a MRI data set that was obtained the day before, the nidus first was determined on stereotactic angiograms in a strictly a.p. and lateral view. The nidus was defined as the planning target volume (PTV). Critical structures were drawn slice by slice using fused high spatial-resolution T1- and T2-weighted MRI data. In a second step, the PTV was modified based on the results of MRI and then re-evaluated with the angiograms. 3-D dose distribution was calculated by BrainSCAN 5.3x until 2008, since 2009 we use iPlanNet/iPlanRT 4.0x; (both BrainLAB®, Feldkirchen, Germany). Conformal treatment plans were designed for all cases using a planning algorithm that involved setting dose constraints to minimize irradiation delivered to critical structures such as optic pathways and the brain stem. 9 conformal non-coplanar static beams in all 3 patients were used. All targets were treated with a single isocenter. The single dose was 20 Gy at the isocenter of the PTV encompassing the 80–90% isodose. Maximum target dose did not exceed 107% and there was no significant radiation dose applied to the organs at risk (OAR). All delivery parameters were according to the guidelines of the Radiation Therapy Oncology Group (RTOG) for stereotactic radiotherapy including a conformity index (CI) between 1.0 and 2.0.

Radiosurgery was well tolerated without acute toxicities within the first 6 months in all cases. Toxicities were evaluated with the Common Terminology Criteria (CTC) for Adverse Events version 3.0. Radionecrosis was defined in accordance with Blonigen et al. as (1) any patient requiring steroid therapy with MRI changes, (2) characteristic MRI changes that persisted on two consecutive scans, (3) MRS findings consistent with necrosis or (4) histologic evidence of necrosis [13].

### Follow-up evaluation

After SRS, follow-up was performed after 6 months, 12 months, and thereafter at intervals of 12 months. Regular follow-up studies included clinical examination, brain MRI, and a single DSA examination 1.5 - 2 years after radiosurgery.

### MRS acquisition and evaluation

^1^H-MRS was performed on 3.0 Tesla whole-body MR systems (Achieva 3.0 TX and Ingenia 3.0 T, Philips Healthcare) using a birdcage quadrature head coil for signal transmission and reception. 2D spectroscopic imaging (2D-SI) was acquired with TR/TE 1750/140 ms at the level of the AVM nidus as an axial slice with 2 cm thickness and 16^2^ matrix elements reconstructed to 20x20 over a field of view (FOV) of 160 mm, thus yielding an in-plane voxel size of 8 mm. Water suppression was achieved by Gaussian excitation pre-pulses, and signal contamination from skull lipids was reduced by restricting spin excitation and refocusing to a PRESS-localized [14] volume of interest (VOI) of 100 x 100 mm centered within the brain. In addition, water-suppressed single-voxel (SV) MRS in a small VOI encompassing the contrast-enhancing part of the lesion was performed with TR 2000 ms and TE140 ms. 2D-SI and SV spectra were sampled with 1024 data points at 2 kHz bandwidth, corresponding to a non-interpolated frequency resolution of 2 Hz/pt, and SV was averaged over 128 signal excitations. In all 3 AVM cases, short echo-time SV with TE 30 ms and 64 signal averages was additionally accomplished to analyse lipid signal components in more detail and to assess spin-coupled metabolites like myo-inositol and glutamate/glutamine. Absolute quantification of metabolite concentrations from the SV spectra was achieved as described in [15,16]. In brief, the NAA signal was referenced to the signal of internal tissue water within the VOI obtained from unsuppressed MRS with TR/TE 3500/80 ms, and metabolite levels were further corrected for partial cerebrospinal fluid (CSF) volume within the VOI. Total measurement time for all 2D-SI and SV acquisitions was less than 20 min.

2D-SI and SV spectra were time-domain quantified applying the AMARES algorithm of the MRUI software package [17,18] after preprocessing with matched Lorentz-Gauss filtering. From the 2D-SI data, signal ratios of Cho/Cr, Cho/NAA, and NAA/Cr were determined for selected spectra within the lesion area (averaged over all contiguous 2D-SI voxels comprising more than 50% of contrast enhancing tissue) and were compared to those for corresponding voxels in the contra-lateral hemisphere, as well as intensity ratios of the respective metabolite in the lesion voxels relative to the contra-lateral voxels. Absolute levels of Cho, Cr, and NAA obtained from the SV measurements in the radiogenic lesion as well as the metabolite ratios were compared to normal values for the respective cerebral region [19].

### Statistical analysis

Statistical analysis was performed using Excel (Microsoft, Seattle, WA, USA) software.

## Results

Radiogenic changes were observed in all patients in follow-up MRI. In one patient, radionecrosis with pronounced blood–brain-barrier disruption and a moderately space-occupying edema was found; this patient was oligosymptomatic and symptoms resolved completely after temporary high-dose cortisone therapy.

Illustrative case presentation with repeated MRS (Patient #1 Tables 1, 2, 3).

**Table 2:** Summary of SRS treatment data, PTV=planning target volume, Dose = single treatment dose in Gy prescribed to the isodose line covering 80 – 90% of the PTV, B=conformal non-coplanar static beams, CTC=Common Terminology Criteria for Adverse Events, version 3.0

**No**	**Nidus=PTV (ccm)**	**Dose (Gy)**	**Technique**	**Nidus occlusion**	**Radionecrosis**	**Adverse events (CTC grade)**
1	2.75	20	9 B	**+**	**+**	transient Broca aphasia 1°
2	6.89	20	9 B	**+**	**-**	none
3	6.41	20	9 B	**+**	**-**	none

**Table 3:** Summary of MRS data obtained from single-voxel (SV) acquisitions and from 2D spectroscopic imaging (2D-SI) in the three patients

**No.**	**Single-voxel MRS (SV) in AVM lesion**					**2D-SI**				
									***contra-lateral***	
	**Cho/Cr**	**NAA/Cr**	**[Cho]**	**[Cr]**	**[NAA]**	**Cho**	**Cr**	**NAA**	**Cho/Cr**	**NAA/Cr**
	***metabolite ratios***		**(mmol/L)**	**(mmol/L)**	**(mmol/L)**	***signal ratios lesion/contra-lateral***			***metabolite ratios***	
1 a	2.08	0.39	1.59	2.86	0.80	0.84	0.32	0.09	1.23	2.15
1 b	2.67	0.46	1.41	1.98	0.65	0.81	0.41	0.22	1.02	1.97
2	1.38	1.05	1.68	5.02	3.78	0.64	0.62	0.34	1.27	2.27
3	1.69	1.23	2.00	4.49	4.01	0.46	0.28	0.26	0.98	1.74
Normal values (from [19])										
Mean	1.11	2.81	2.07	6.99	14.51					
*SD*	*0.16*	*0.29*	*0.40*	*0.85*	*1.42*					

In the first column, 1a and 1b denote the initial and the second (4 months later) therapy follow-up MRI/MRS examination of patient #1, respectively. In the next columns, Cho/Cr and NAA/Cr represent metabolic ratios determined from the SV spectra at TE 140 ms, and the absolute concentrations of choline, creatine, and NAA as derived from the SV acquisitions in the lesion are listed. The 2D-SI results comprise the ratios of the signal intensity of the respective metabolite within the AVM lesion relative to that in the corresponding voxels in the unaffected contra-lateral hemisphere, and in the last two columns the metabolite ratios Cho/Cr and NAA/Cr measured in the contra-lateral voxels are shown. Below the patient results, normal values for relative and absolute metabolite levels in frontal/parietal white matter, taken from [19] are given for comparison.

**Figure 1:**
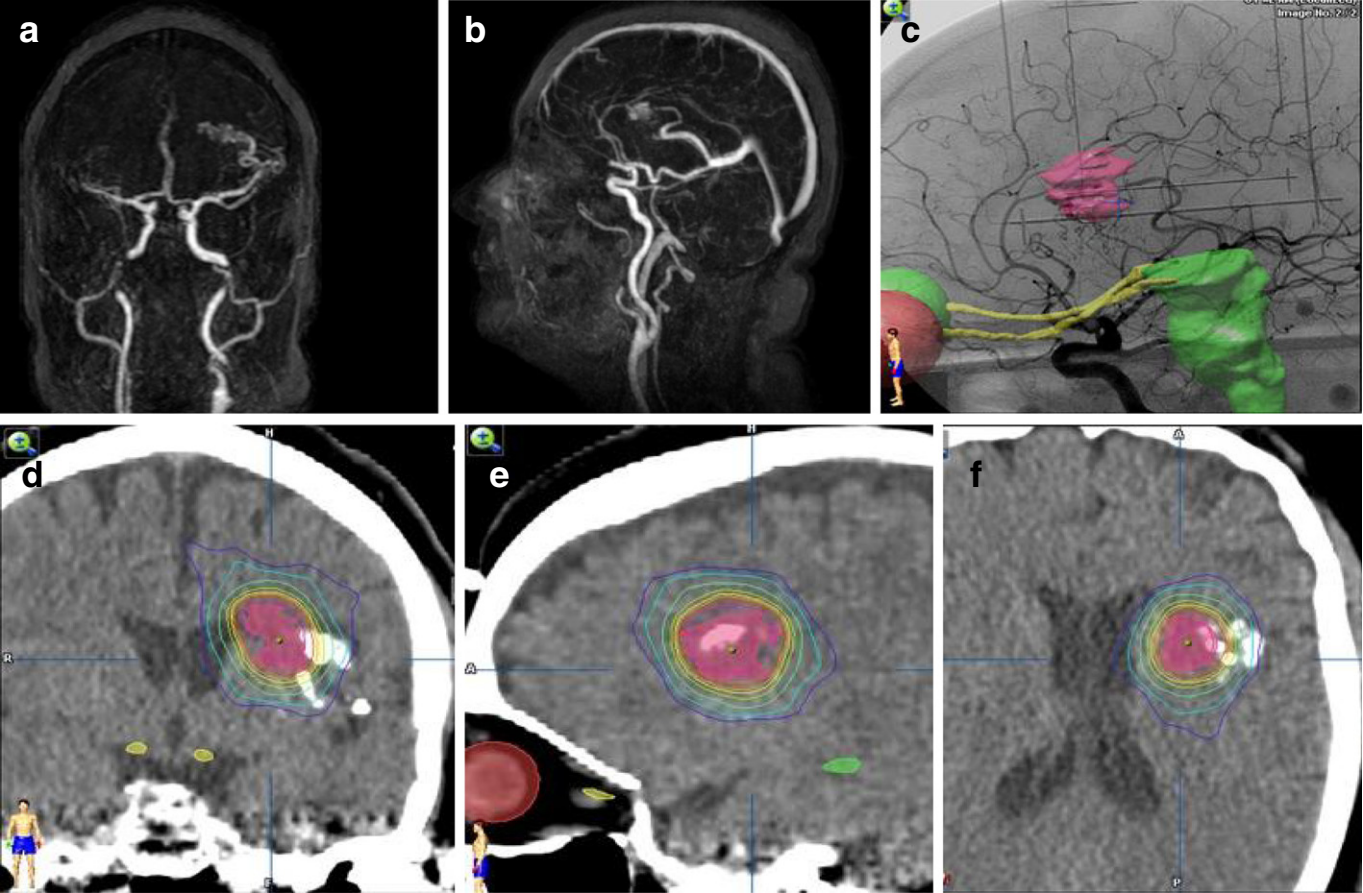
**Incidental left frontal cerebral arteriovenous malformation in a 65 year-old woman after 3 partial embolizations (patient #1).** Maximum intensity projections of pre-operative dynamic 4D-MR angiography (**a**: frontal view, **b**: saggital view), treatment planning (**c**: fused DSA and planning target volume (PTV), **d**-**f**: fused CT with PTV and isodose lines).

**Figure 2:**
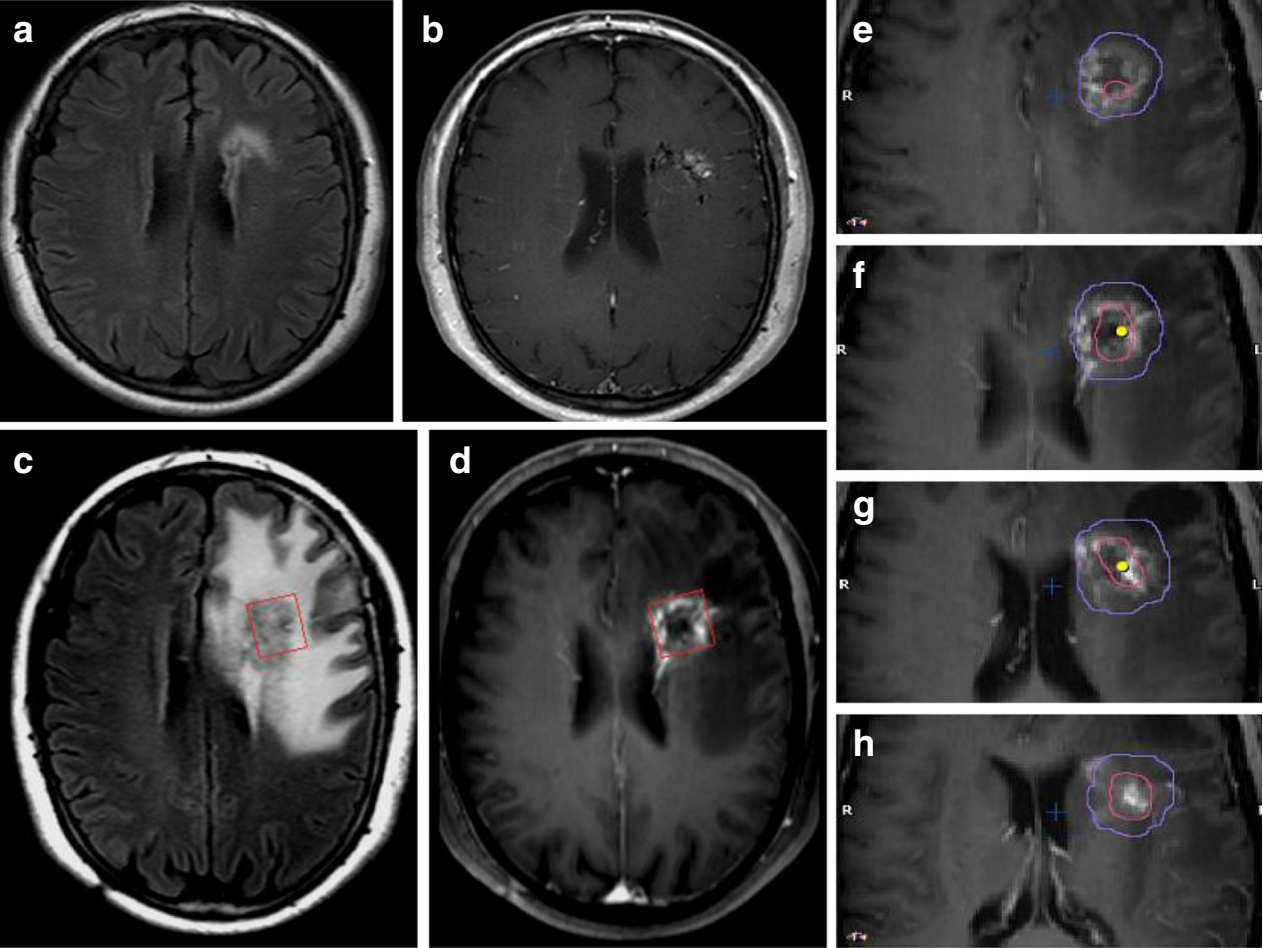
**Same patient as Figure **1**.** Pre- and post-treatment MRI (FLAIR: **a**,**c**; contrast-enhanced MRI: **b**,**d**), **e**-**h**: fused post-treatment MRI). Red frame in **c**,**d** indicates selected volume for SV-MRS. Red line in **e**-**h** corresponds to PTV, purple line in **e**-**h** represents 10 Gy isodose line.

**Figure 3:**
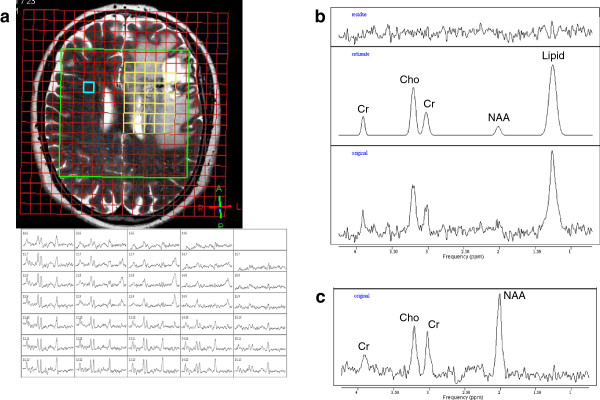
**Same patient as Figure **1. MR spectroscopy (TE 140 ms) in first post-treatment examination. **a**: Display of 2D-SI grid (red) over FOV of 160 mm, PRESS-localized VOI of 100 mm (green frame), spectra selection grid (yellow) on T2-weighted MRI (top), and of 34 selected 2D-SI spectra (below) from the lesion area arranged in a pattern corresponding to the yellow grid. **b**: SV-spectrum from a 9 mL-VOI (red frame in Figure 2c,d) in AVM lesion shows strongly reduced Cr, barely visible NAA, and a high lipid peak. **c**: 2D-SI spectrum from healthy tissue contralateral (blue-framed grid element in **a**) to the AVM.

**Figure 4:**
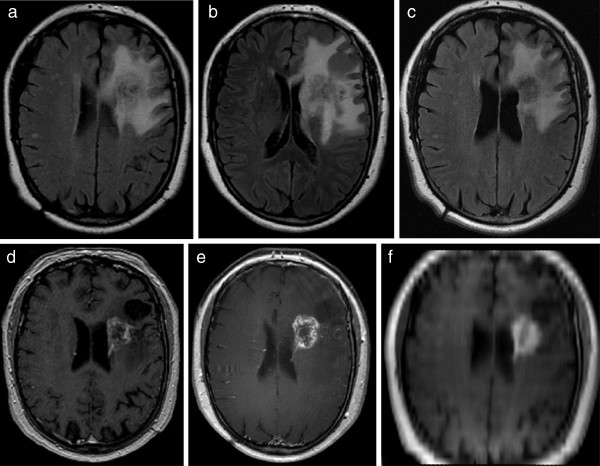
**Same patient as Figure **1**.** Additional follow-up post-treatment examinations reveal constant hyperintensities in FLAIR (**a**-**c**) and contrast-enhanced MRI (**d**-**f** [f = axial MPR]) six weeks (**a**,**d**), three more months (**b**,**e**), and 20 months (**c**,**f**) after initial post-treatment MRI as shown in Figure 2 indicating non-malignant disease.

An incidental left frontal arterio-venous-malformation (AVM) was found in a 62 year old female who presented with unrelated vision impairment. The AVM was related to the internal capsule and Broca area and showed no signs of former bleeding. After DSA and WADA testing it was classified as Spetzler Martin grade III. Microsurgical resection was considered too riskful; therefore it was treated with 3 partial embolizations followed by radiosurgery. All delivery parameters were according to the guidelines of the RTOG for stereotactic radiotherapy including a CI of 1.68 (Figure 1). 16 months after radiosurgery she developed aphasia. Under steroid therapy, a complete remission was achieved. Follow up MRI including temporally-resolved MR angiography revealed a complete elimination of the nidus, but at the same time showed a ring enhancement and a marked perifocal edema (Figure 2). Additional MRS in the enhancing area measured a high ratio of Cho to Cr (> 2.0) suspicious for malignancy, although absolute concentration of Cho was not increased (Figure 3). In MRI control after 4 and 20 months, ring enhancement was unchanged and the perifocal edema showed slight regression (Figure 4). MRS revealed a stable metabolic pattern over a time course of 4 months with persistent high Cho/Cr, extremely low NAA concentration < 1 mmol/L, and strongly decreased Cr levels < 3 mmol/L. Taking into account that our patient requires steroid therapy, that the MRI changes were stable on more than two consecutive scans, were only slightly regredient over a 20 months period and did not reverse completely, and that the repeated MRS findings finally were consistent with necrosis, we scored this case as radionecrosis.

### MRS results

**Figure 5:**
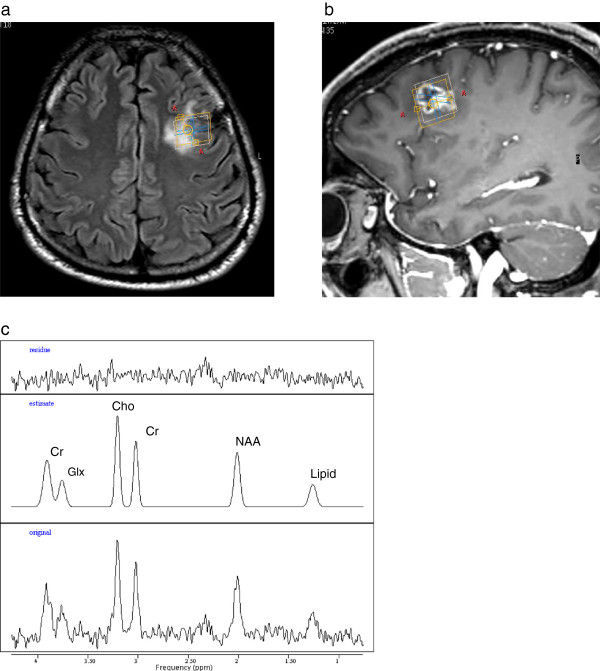
**MRS in AVM patient (#2) with normal post-interventional state.** Radiogenic changes after treatment are seen as hyperintensities in axial FLAIR (**a**) and sagittal contrast-enhanced MRI (**b**). **c**: SV-spectrum from 6 mL-VOI (yellow frame in **a**, **b**) in AVM lesion reveals a moderately increased Cho/Cr ratio of 1.38 and smaller signal from lipids.

Table 3 summarizes for each MRS examination of the patients the relative and absolute concentrations of Cho, Cr, and NAA obtained from SV-MRS in the lesion, as well as the ratios of the metabolite levels within the AVM lesion measured by 2D-SI (averaged over all spectra from the contrast-enhancing area) relative to those of corresponding voxels in the unaffected contra-lateral hemisphere (side comparison). Further, metabolite ratios determined for the unaffected contra-lateral voxels and normal metabolite concentrations [19] in the respective cerebral region are included for comparison. The choline / creatine ratio in the lesion of the 2 patients without radionecrosis remained below 1.7 and was considerably smaller than in AVM patient #1 with radionecrosis, and in the 2D-SI side comparison the choline level in the lesion was less than 65% of the contra-lateral value while in the patient with radionecrosis the relative Cho level reached about 80-85%. Nevertheless, all cases revealed considerably lower concentrations of metabolites in the lesion site. In the 2D-SI and SV spectra from the AVM lesion of the patient with radionecrosis a high peak from (phospho) lipids was observed even at the long TE 140 ms (Figure 3), whereas the lipid signal was not or only slightly increased in the two patients with normal post-interventional state (Figure 5).

## Discussion

Our study contributes to the understanding of radionecrosis in radiosurgically treated AVM. Radionecrosis, being a calculated reaction in tumor radiosurgery, has to be avoided strictly in AVM radiosurgery. In this situation the goal is to obliterate the AVM blood vessels, because radionecrosis of brain may involve excessive undesirable side effects including perifocal edema and mass effect. Our data may be a valuable contribution to the better understanding of radiogenic changes after radiosurgery in MRS studies and differentiate them from primary or secondary brain malignancies.

Changes after radiotherapy may be extremely complex. Irradiated tissues may undergo several coexisting processes, including progressive cellular necrosis and gliosis, inflammatory changes, and reactive glial cell proliferation. In malignant lesions, coexisting radiation-induced changes and residual tumor have also been observed. As a consequence, the interpretation of MRS and MRI may also be complex. For instance, tumor recurrence after high dose RT likely additionally contains both tumor and necrosis components. Furthermore, in a heterogeneous lesion of both tumor and necrosis after RT, either single-voxel or multi-voxel MRS may not capture the tumor cluster due to partial volume averaging [8].

In our study, for the first time we were able to extensively study radiogenic changes including those in MRS in radionecrosis in non-tumor patients; therefore, influencing factors like tumor progression or the presence of an infiltrative zone as they are typically observed in gliomas, are eliminated in our patients. This results in the unique opportunity to study MRS changes in vivo that are likely a result of almost pure radionecrosis in non-tumorous brain tissue, especially since there was no former bleeding with potential brain damage in any of the cases.

We identified the following metabolic patterns associated with radiation necrosis:

• In regions with coexisting cellular necrosis and reactive mechanisms (normal post interventional state), no increase in Cho and a Cho/Cr ratio < 2.0 was found.

• In regions with predominantly cellular necrosis (radionecrosis), increased lipid levels, but unchanged or slightly decreased levels of Cho and extremely reduced NAA and Cr levels were found with a markedly increased Cho/Cr ratio > 2.0.

The embolization material was analyzed separately using MRS in vitro. Because of the lack of material-specific lipid peaks at the in-vivo MRS examinations, we could unambiguously identify radiogenically induced phospholipid peaks.

In this study, MRS did perform as well as previously reported for differentiating radiation injury from malignant tumor [6-8]. However, prior publications were primarily focused on observations in glioma patients after fractionated RT or on brain metastases treated with high doses of single fraction radiosurgery [6-8].

In the literature, Cho concentration and the Cho/Cr ratio are considered to be the most useful markers for the differentiation of neoplasms from radiation necrosis in MRS. Spectra with distinctly elevated absolute Cho levels and/or Cho/Cr > 2.0 are considered suggestive of a malignant tumor [8]. Our cases demonstrate the importance of careful consideration of the absolute values of the individual metabolites, especially the Cr level, because a necrosis-induced strong reduction in Cr may likewise lead to large Cho/Cr ratios. Furthermore, it has to be kept in mind, that the known cut-off value for Cho/Cr is appropriate for most parts of the frontal and parietal brain, but will be too high for lesions in cerebral regions with naturally low Cho concentrations, for instance in the occipital lobe [20].

MRS has a published sensitivity of 81% and a specifity of 71% for differentiation between recurrent tumor and radiation necrosis [21]. Results from MRS may be comparable to those from FDG-PET or SPECT. [18F]FDG-PET has a sensitivity of 75% and a specificity of 81% for the detection of recurrent tumor versus radiation necrosis [22]. Moreover, in patients after stereotactic radiotherapy for brain metastasis, co-registration of [18F] FDG-PET images with MRI yields an improvement of the sensitivity for the detection of recurrent tumor from 65 to 86% [23]. In the recent years [11C] MET-PET has shown to be successfull in differention between recurrent tumor and radiation necrosis [24,25], similar results have been obtained with other tracers [26,27]. Foroughi et al. reported some cases of AVM after radiosurgery with false positive results suggestive for malignancy and recommended caution when attempting to differentiate radionecrosis from neoplasia using SPECT scans [11].

Finally, amide proton transfer imaging in 7 Tesla MRI maybe an alternative and possibly promising approach, first results concerning radionecrosis after AVM radiosurgery were recently published by Gerigk et al. in 2012 [28], but the applied technology and the required MR equipment are highly challenging and available only to very few clinical centers, and its usefulness has still to be evaluated in larger patient samples.

In our opinion, imaging techniques like MRS and PET/SPECT or APT imaging cannot alone prove a radionecrosis, they should not be regarded as competing but as complementary methods, and they can only be a supplement to conventional imaging and have always to be evaluated in context with the clinic.

In clinically and by MRI imaging doubtful cases, MRS in our opinion provides useful information and just helps in some cases to avoid a possibly unnecessary invasive procedure such as re-operation or biopsy. With hints for radionecrosis the duration of high-dose antiedematous dexamethasone therapy maybe will be shorter, because beside strong MRI changes most patients with radionecrosis are a- or oligosymptomatic and a long-time cortisone therapy is not necessary. In some special cases instead of high-dose cortisone a more specific therapy with the monoclonal vascular endothelial growth factor (VEGF) antibody bevacizumab may be useful. In 2011 for the first time at a larger scale, Levin et al. have described a therapy effect of bevacizumab in radiation necrosis confirming the relative new theory that blocking VEGF at an early stage with bevacizumab could be an option to reduce the development of radiation necrosis by decreasing the vascular permeability [29].

### Limitations

Due to the relatively large required voxel size for SV-MRS, partial volume averaging over tissue with a mixture of coexisting processes like cellular necrosis and cell proliferation represents significant obstacles in the diagnostic accuracy of MRS. Spectroscopic imaging allows better spatial resolution, but at the cost of reduced signal-to-noise, longer measurement time and increased artifact sensitivity.

Our small sample size does not allow for generalization of our results and must be confirmed by further studies with larger sample size. However, the unique opportunity to obtain spectroscopic data of a lesion, which is imaging-wise suspicious for malignancy after radiation therapy of a benign lesion and the detection of the importance of not concentrating on the metabolite ratios alone, but to carefully take the absolute concentrations into account, are important findings that need to be carefully regarded in interpretation of spectroscopic results.

## Conclusions

We were able to extensively examine radiogenic changes (radionecrosis) by means of MR spectroscopy in AVM patients treated with radiosurgery. In contrast to other studies, for the first time we systematically investigated non-tumorous lesions avoiding overlays by tumor necrosis; this enabled us to study (with minor restrictions) radionecrosis after radiosurgery in its pure form and specific patterns of MRS spectra regarding radionecrosis were identified; however, the results can be misleading and must be interpreted with caution in context with the clinic to avoid misdiagnosis. MRS is a supplement to the conventional imaging, and no therapy decision should be based solely on MRS data.

## Abbreviations

MRI: Magnetic resonance imaging; MRS: Magnetic resonance spectroscopy; MRA: Magnetic resonance angiography; CT: Computed tomogram; cAVM: Cerebral arteriovenous malformation; SM: Spetzler-Martin grade; RT: Radiotherapy; DSA: Digital subtraction angiograpy; Gy: Gray; OAR: Organs at risk; PTV: Planning target volume; RTOG: Radiation Therapy Oncology Group for stereotactic radiotherapy; CI: Conformity index; CTC: Common Terminology Criteria for adverse events; 2D-SI: 2D spectroscopic imaging; FOV: Field of view; SV: Single-voxel; VOI: Volume of interest; TR: Repetition time; TE: Echo time; CSF: Cerebrospinal fluid; Cho: Choline compounds; NAA: N-acetyl aspartate; Cr: Total creatine (creatine + phosphocreatine); VEGF: Vascular endothelial growth factor.

## Competing interests

The authors declare that they have no competing interests.

## Authors’ contributions

All of the authors have been involved in drafting this paper and have read and approved the final manuscript. JB conceived the idea of the paper, performed the literature research and statistical analysis, wrote the paper, performed the radiosurgical procedures, DH with supervision of WW performed the MRI scans including MRA and wrote the part of the paper concerning the illustrations, FT with considerable help of WB was the physicist who performed the MRS and wrote the part of the paper concerning the MRS method and its results as well as performed with JB the relevant literature research. HS as the chief radiologist and supervisor of the clinic revised the final manuscript.
